# Adolescents at risk of eating disorders: The mediating role of emotional distress in the relationship between differentiation of self and eating disorders

**DOI:** 10.3389/fpsyg.2022.1015405

**Published:** 2023-01-11

**Authors:** Ora Peleg, Meyran Boniel-Nissim, Orna Tzischinsky

**Affiliations:** Max Stern Academic College of Emek Yezreel, Emek Yezreel, Israel

**Keywords:** eating disorders, adolescents, emotional distress, stress, anxiety, depression, differentiation of self

## Abstract

**Introduction:**

Adolescents may feel dissatisfied with their bodies, which may lead to a risk of eating disorders (EDs) due to several factors, with emotional distress being one of the most important. Evidence suggests that family might be one of the most significant factors that may increase or decrease emotional distress. An important family pattern found to contribute to mental and physical health is the differentiation of self (DoS). The primary purpose of the current study was to map the complex relationships between DoS, emotional distress, and EDs among adolescents. We hypothesized that emotional distress would mediate the relationship between DoS and the risk of EDs among adolescents. Moreover, based on findings indicating a higher risk of EDs among females, we expected sex differences in the research indices and the mediation model.

**Methods:**

The sample included 194 non-clinical adolescents (mean age 15.15; mean BMI 21.66). Preliminary analyses examined differences between males and females using *t*-tests. In addition, Pearson correlations were run to assess the association between background variables and the study metrics among males and females. To examine the mediation effect, we ran SEM.

**Results:**

Due to a sex moderation effect, two mediation models were run (SEM), one for females and one for males. Results indicated that emotional distress partially mediated the relationships between DoS and the risk of EDs. In addition, sex differences were found in the mediated indices, showing that among female adolescents, perfectionism is the only dimension of EDs that was associated with DoS through the mediation of emotional distress. While the relationship between emotional distress and the risk of EDs is well documented.

**Conclusions:**

It is concluded that high DoS may reduce emotional distress, which may, in turn, decrease the risk of EDs. In addition, the results enable an in-depth understanding of specific risk factors of EDs that characterize each sex.

## 1. Introduction

Adolescence is characterized by accelerated changes occurring in various areas (Sawyer et al., 2018). For example, in the family, teenagers take more responsibility and go through a process of individuation and separation from the family unit. Similarly, at school, academic demands increase, and in the wider society, they explore new experiences with peers and engage in new adult activities (Helsen et al., 2000; Steinberg, 2007). Physically, adolescents experience changes that shape their perceptions of themselves and their attitudes toward their appearance and may lead to dissatisfaction with their body image (Markey, 2010; Chulani and Gordon, 2014; Voelker et al., 2015).

Negative body perception was found to predict weight control behavior that may be manifested in harmful actions, such as fasting, purging, extreme diets, or intense exercise (Knowles et al., 2009). In addition, body dissatisfaction and preoccupation with weight were found to be related to eating disorders (EDs; Voelker et al., 2015). EDs are well-defined by the American Psychiatric Association’s *Diagnostic and Statistical Manual of Mental Disorders* 5th Edition (DSM-V) as diseases characterized by severe disturbances in eating behaviors. According to the DSM-V, the following EDs are recognized: anorexia nervosa (AN), atypical anorexia nervosa (AAN), bulimia nervosa (BN), binge eating disorder (BED), and avoidant/restrictive food intake disorder (ARFID; the Diagnostic and Statistical Manual of Mental Disorders, 5th Edition, DSM-5, American Psychiatric Association, 2013).

A significant limitation in the current knowledge is that most studies that examined the detection, treatment, and course of EDs have focused on females, leading to sex bias (Silén et al., 2021). Thus, it was found that body image disorder was more common among girls (15%) than boys (6.8%; Quiles-Marcos et al., 2011). Recently community studies found the prevalence of EDs ranged from 3.1 to 17.9% among females and 0.6 to 2.4% among males (Silén et al., 2020). In addition, according to Silén et al. (2021), the rate of people suffering from EDs diagnosed according to the DSM-5 criteria is 1:10 male/females, respectively, in clinical samples and 1:4 in non-clinical samples. Yet it should be noted that recent studies show that the prevalence of EDs among men is increasing. Therefore it is recommended to further examine this issue among male adolescents for identification, detection, and tailored treatment (Nagata et al., 2020).

Previous studies yielded positive associations between EDs and stress (e.g., Aoun et al., 2013; Solmi et al., 2020), anxiety, and depression (e.g., Drieberg et al., 2019) among adolescents. Teenagers’ coping with these pressures alongside the many changes that occur during adolescence is challenging and requires strength and personal qualities that will enable them to successfully deal with the various difficulties and stresses (Orkibi and Ronen, 2015).

There is evidence that family might be one of the most significant factors in increasing or decreasing emotional distress. Indeed, it was observed that family patterns both contributed to mental and physical health and increased the risk of EDs (Erriu et al., 2020). According to Bowen’s (1978) Family Systems Theory, the emotional, physiological, and behavioral patterns in the family system are passed from generation to generation. Bowen argued that childhood and adolescence are critical periods for achieving developmental tasks and regulating emotions. This is a time when their personality is in a process of formation and it is still possible to help prevent critical difficulties (Kerr and Bowen, 1988). During this period, adolescents learn to be less dependent on their parents and develop symmetrical relationships with them, thus increasing their tendency to make decisions independently (Bowen, 1978; Kerr and Bowen, 1988).

One of the most important family patterns that has been derived from this theory is differentiation of self (DoS). DoS is a pattern passed down through the generation. It describes, on an interpersonal level, the ability to balance intimacy and autonomy in relationships with significant others, and on the intrapersonal level, the ability to balance cognition and emotion. For adolescents, DoS includes six components: own emotional reactivity, maintaining a clear identity, hypersensitivity to others, seeking emotional distance, emotional dependence on others, and reactive distancing from a close friend (Knauth and Skowron, 2004). DoS takes shape during childhood and adolescence and is influenced by the parents’ level of DoS. High DoS allows an individual to adhere to his feelings, thoughts, and needs and to deal more effectively with stressful situations and social pressures, while low DoS increases the difficulty of behaving authentically, regulating emotions, and coping with stress and crises.

Indeed, it was demonstrated that low levels of DoS are associated with high levels of anxiety in adolescents (Peleg et al., 2015), depression (Choi and Murdock, 2017), problematic coping styles, stress, physical health problems (Hooper and Doehler, 2011) and EDs (Peleg et al., 2022). Thus, for example, Doba et al. (2018) found in a sample of adolescents that low DoS may lead to distortions in self-perception, channel emotions into excessive eating or extreme weight gain, and increase pathologies aimed at improving body image.

It has been suggested that emotional distress may increase the risk of EDs, but this depends on certain family indices (e.g., Peleg et al., 2022). Hence, it is essential to examine its mediating role between the two variables of DoS and the risk of EDs. Thus, for example, in a recent study conducted among young adults (Peleg et al., 2022), emotional distress was found to mediate the relationship between DoS and the risk of EDs and specifically, between I-position and the risk of EDs, showing that a low level of I-position indicates a tendency to avoid direct communication in interpersonal relationships. This leads to increased psychological distress, which may, in turn, increase predisposition to EDs. However, the natural course of DoS, emotional distress, and the risk of EDs among adolescents remains poorly understood and thus merits further investigation.

## 2. The current study

Given the evidence of both associations between emotional distress and EDs and emotional distress and DoS and the mediating role of emotional distress between these variables, the primary purpose of this study was to map the complex relationships between these factors. Despite identifying DoS as a significant developmental phenomenon and its association with psychological and physiological outcomes, investigating how emotional distress mediates the relationships between DoS and the risk of EDs has been negligible. The current study expanded upon the relationships of the variables under study by examining the nature of this critical pathway. In addition, since this issue has not yet been examined among adolescents, we aimed to address this knowledge gap. Hence, we hypothesized that emotional distress (stress, anxiety, depression) would mediate the relationship between DoS (own emotional reactiveness, maintaining a clear identity, hypersensitivity to others, seeking emotional distance, emotional dependence on others, and reactive distancing from a close friend) and the risk of EDs (drive for thinness, bulimic tendencies, body dissatisfaction, and perfectionism) among adolescents. In addition, given that EDs are one of the most gendered mental health disorders (Weber et al., 2019), there is great importance in investigating the differences between male and female adolescents to learn more about the risk factors of EDs. Therefore, our second aim was to examine sex differences in the research indices and the mediation model.

## 3. Materials and methods

### 3.1. Participants

The number of participants in the current study was 194 adolescents, of whom 129 were females (66.5%) and 65 males (33.5%; Table 1). Participants’ ages ranged from 12 to 20 (*M* = 15.14, SD = 1.71), and their BMI ranged from 15.01 to 33.15 (*M* = 21.66, SD = 3.54). The inclusion criterion was adolescents whose parents were married or living together. This criterion is based on Bowen’s (1978) assumption that stressful life events (e.g., divorce) may change DoS levels (e.g., Peleg, 2014). We therefore chose not to include participants whose family status could bias the results.

**Table 1:** Characteristics of study participants (*N* = 194).

	*n*	%	*M*	SD	Range
**Sex**					
Females	129	66.5			
Males	65	33.5			
Age			15.14	1.71	12–20
BMI			21.66	3.54	15.01–33.15

### 3.2. Research instruments

*A Personal Information Questionnaire* was specifically constructed for the present study. The instrument includes background information, e.g., religion, age, sex, weight, and height.

*The Eating Disorder Inventory-2 (EDI-2)* (Garner, 1991) is a multidimensional and practical questionnaire for clinical and non-clinical purposes (Lee et al., 1998). The EDI-2 contains 91 items, rated on a six-point scale, and 11 sub-scales. Based on previous studies (Latzer et al., 2018), we used in the current research four significant sub-scales tapping EDs (EDI-2): drive for thinness (DT); bulimic tendencies (B); body dissatisfaction (BD); and perfectionism (P). The EDI-2 was found valid and reliable in a wide range of research studies and has been translated into many languages, including Hebrew (Niv et al., 1998). In the current study, the internal reliability of the questionnaire was good: drive for thinness (*α* = 0.80); bulimic tendencies (*α* = 0.83); body dissatisfaction (*α* = 0.87); and perfectionism (*α* = 0.75).

*Differentiation of Self-Inventory for Adolescents* (Knauth and Skowron, 2004). The original Questionnaire (DSI-R, Skowron and Schmitt, 2003) was adapted for adolescents and translated into Hebrew (Peleg and Harish, 2022). The questionnaire consists of 25 items that assess relationships in general and in the family of origin. The instrument includes six sub-scales: own emotional reactiveness, maintaining a clear identity, hypersensitivity to others, seeking emotional distance, emotional dependence on others, and reactive distancing from a close friend. A sample item: “Closest friend wants too much from me” (reactive distancing from a close friend). The answers are rated on a Likert scale from 1 (completely incorrect) to 6 (completely true). The calculation is based on means scores. The internal reliabilities of the questionnaire in the current study were: for own emotional reactiveness–0.86, for maintaining a clear identity–0.81, for hypersensitivity to others–0.70, for seeking emotional distance-off–0.86, for emotional dependence on others–0.81, and for reactive distancing from a close friend–0.82.

*The Depression Anxiety Stress Scales (DASS-21)* (Henry and Crawford, 2005). This questionnaire is a short version (21 items) of a 42-item self-report instrument designed to measure three related negative emotional states: depression, anxiety, and tension/stress. This measure was translated and adapted into Hebrew and was found reliable and valid. The current study’s internal consistency (Cronbach’s alpha) was good, as the scale was 0.90 (Alon-Tirosh et al., 2021). It includes three sub-scales, depression (7 items), anxiety (7 items), and stress (7 items). Sample item: “I felt that life was meaningless” (depression). Scores are summed such that higher scores indicate more depression, anxiety, or stress. In the present study, we used the total DASS score because there were strong correlations between the three metrics: stress, anxiety, and depression. Factor analysis for the three scales extracted only one component, Eigenvalues = 2.37. Total Variance Explained = 78.88. Internal consistency in the current study was high; the total score was 0.94, for depression 0.89, for anxiety 0.86, and for stress 0.86.

### 3.3. Procedure

After receiving approval from the College Institutional Review Board (on 9.12.2019; EMEK YVC 2020-13) and from the Ministry of Education, we approached school principals in northern Israel. The research assistants received approval from two Jewish public high school principals to participate in the study. Participants who gave their parents and their active consent were allowed to participate in the study (10.8%). The research assistants provided participants with the instructions for filling out the questionnaire: they explained that completing the questionnaires was voluntary, participation in the study allowed complete anonymity, there were no right or wrong answers, and withdrawal from the study at any time was possible. All participants signed an informed consent form. Filling out the questionnaire was done in paper and pencil format. Filling out the questionnaires took about 30 min. No compensation was given for participating in the study.

### 3.4. Data analysis

Preliminary analyses examined differences between males and females using *t*-tests and Chi-square. According to Bonferroni’s correction of 11 comparisons, the significance level was α = 0.00465. In addition, Pearson correlations testing relationships between age, BMI, and study variables were calculated for males and females separately. Since, in the present study, very high correlations were found between the three scales of DASS-21 (depression, anxiety, and stress), a problem arose in the AMOS model. Therefore, to avoid multicollinearity, we used the total DASS-21 score. To test the mediation hypothesis, path analysis was conducted using IBM AMOS. We used multigroup analysis to examine differences between males and females in the different paths. Multigroup analysis in structural equation modeling (SEM) is another form of moderation analysis using grouping variables (In our study, males and females).

## 4. Results

### 4.1. Preliminary analyses

Table 2 presents *t*-tests for examining differences between males and females in the study variables. In addition, a Chi-square test was conducted to examine differences between males and females in the risk of EDs (high risk was determined by a score higher than 14 on the drive for thinness scale). The examination of differences in DoS shows that females are significantly higher in their emotional reactiveness, hypersensitivity to others, and emotional dependence on others but significantly lower in maintaining a clear identity compared to males. No significant differences were found in seeking emotional distance and reactive distancing from a close friend. In addition, females reported significantly higher levels of depression, anxiety, and stress (DASS Score). Finally, females reported significantly higher levels of drive for thinness, body dissatisfaction, and bulimia (EDI-2) than males. In addition, a higher percentage of females than men had a high level of risk of developing EDs. No significant differences were found in perfectionism (EDI-2).

**Table 2:** Differences in the study variables by sex (*N* = 194).

	Gender						*t* (192)	Adj *p*
	Males (*n* = 65)			Females (*n* = 129)				
	*M*	SD	*R*	*M*	SD	*R*		
Differentiation of self-inventory (DSI)
Own emotional reactiveness	2.63	1.24	1.00–5.17	3.52	1.33	1.00–6.00	−4.52	<0.001
Maintaining a clear identity	4.63	1.14	1.00–6.00	4.11	1.06	1.00–6.00	3.10	0.001
Hypersensitivity to others	3.17	1.64	1.00–6.00	4.29	1.35	1.00–6.00	−4.74	<0.001
Seeking emotional distance	2.81	1.70	1.00–6.00	2.87	1.69	1.00–6.00	−0.23	0.407
Emotional dependence on others	2.66	1.30	1.00–6.00	3.02	1.23	1.00–6.00	−1.89	0.030
Reactive distancing from close friend	2.27	1.48	1.00–6.00	2.20	1.29	1.00–6.00	0.37	0.357
DASS scale	0.87	0.70	0.00–2.95	1.05	0.66	0.00–2.81	−1.81	0.036
Eating disorder inventory (EDI)
Drive for thinness	17.63	9.70	7–42	23.36	10.62	7–42	−3.65	<0.001
≤ 14	*n* = 27 (41.5%)			*n* = 35 (27.1%)			$\chi^{2}$ (1) = 4.13	*p* = 0.042
> 14	*n* = 38 (58.5%)			*n* = 94 (72.9%)				
Body dissatisfaction	22.08	10.88	9–49	28.92	10.51	9–54	−4.23	<0.001
Bulimia	14.17	7.20	7–40	17.02	7.50	7–42	−2.54	0.006
Perfectionism	23.31	6.85	9–36	23.02	6.70	11–36	0.28	0.391

Significant levels shown in the table were corrected using Bonferroni correction (*α* = 0.00465).

To examine the associations between the study variables, Pearson correlations were run among males and females separately (Table 3). As can be seen in Table 2, own emotional reactiveness, hypersensitivity to others, seeking emotional distance, emotional dependence on others, and reactive distancing from a close friend were positively associated with DASS-21, and maintaining a clear identity was negatively associated with DASS (these are the associations between the independent variables and the mediating variable in the current study). In addition, DASS-21 was positively associated with the four scales of EDI-2 (These are the associations between the mediating variable and the dependent variables).

**Table 3:** Correlations between study variables by sex (*N* = 194).

	1	2	3	4	5	6	7	8	9	10	11	12	13
1. Age	-	0.30^**^	0.14	0.02	0.23^*^	0.16	0.12	0.18	0.13	0.01	0.09	0.21^*^	−0.07
2. BMI	0.04	-	0.14	−0.14	0.15	0.07	0.12	0.25^*^	0.16	0.34^**^	0.54^**^	0.35^**^	−0.10
3. Own emotional reactiveness	−0.01	0.08	-	−0.24^*^	0.69^**^	0.38^**^	0.73^**^	0.58^**^	0.77^**^	0.56^**^	0.49^**^	0.57^**^	0.33^**^
4. Maintaining a clear identity	0.17^*^	−0.01	−0.26^**^	-	−0.07	−0.04	−0.25^*^	−0.18	−0.29^**^	−0.21^*^	−0.45^**^	−0.26^*^	0.05
5. Hypersensitivity to others	0.10	0.02	0.65^**^	−0.19^*^	-	0.48^**^	0.72^**^	0.45^**^	0.71^**^	0.55^**^	0.46^**^	0.51^**^	0.38^**^
6. Seeking emotional distance	−0.01	0.01	0.13	0.09	0.17^*^	-	0.40^**^	0.52^**^	0.61^**^	0.29^**^	0.40^**^	0.60^**^	0.33^**^
7. Emotional dependence on others	0.02	0.16^*^	0.53^**^	−0.35^**^	0.49^**^	0.32^**^	-	0.53^**^	0.72^**^	0.49^**^	0.40^**^	0.51^**^	0.37^**^
8. Reactive distancing from close friend	0.01	0.18^*^	0.29^**^	−0.08	0.20^*^	0.46^**^	0.39^**^	-	0.56^**^	0.55^**^	0.54^**^	0.69^**^	0.28^*^
9. DASS	−0.10	0.13	0.54^**^	−0.34^**^	0.39^**^	0.33^**^	0.54^**^	0.20^*^	-	0.55^**^	0.61^**^	0.69^**^	0.33^**^
10. Drive for thinness	0.00	0.38^**^	0.38^**^	−0.38^**^	0.32^**^	0.16^*^	0.38^**^	0.31^**^	0.26^**^	-	0.75^**^	0.62^**^	0.34^**^
11. Body dissatisfaction	−0.07	0.36^**^	0.30^**^	−0.43^**^	0.27^**^	0.20^*^	0.43^**^	0.31^**^	0.29^**^	0.63^**^	-	0.68^**^	0.06
12. Bulimia	0.02	0.24^**^	0.26^**^	−0.35^**^	0.11	0.12	0.25^**^	0.24^**^	0.22^**^	0.50^**^	0.32^**^	-	0.34^**^
13. Perfectionism	−0.11	0.08	0.28^**^	−0.12	0.18^*^	0.11	0.19^*^	0.13	0.26^**^	0.32^**^	0.14	0.30^**^	-

^*^p < 0.05, ^**^p < 0.01.

The correlations appearing above the diagonal were calculated among males (*n* = 65), the correlations appearing below the diagonal were calculated among women (*n* = 129).

### 4.2. Path analysis model

Multigroup path analysis was conducted to explore the potential moderating role of sex in the hypothesized mediation model. According to our hypothesis, the association between DoS and EDs would be mediated by depression, anxiety, and stress (DASS). The results showed a significant decrease in the goodness of fit indices in the constructed model (which constructs the model paths so that they are equal among males and females) compared to the unconstructed model (which separates the path for males and females), $\chi^{2}$ (15) = 30.27, *p* = 0.011. In other words, these results indicate that the goodness of fit indices of the model that calculates separate paths for males and females are better, $\chi^{2}$ (38) = 1.39, *p* = 0.055, GFI = 0.96, CFI = 0.98, RMSEA = 0.045, compared to model for males and females together, $\chi^{2}$ (53) = 1.57, *p* = 0.005, GFI = 0.93, CFI = 0.97, RMSEA = 0.054. Based on the results, separate models will be presented. According to modification indices, we added direct paths between DoS and EDs. In addition, BMI was entered as a covariate. See Table 4 for standardized parameters for the final model.

**Table 4:** Path analysis summary by sex.

Exogenous variables	Endogenous variables	Direct effect		Differences between paths by sex	Female		Male		
		Beta	*p*	Beta	*p*	Chi (1)	*p*
Own emotional Reactiveness	DASS	0.40	<0.001	0.40	<0.001	0.19	0.667
Maintaining a clear identity		−0.20	0.005	−0.14	0.034	0.47	0.493
Hypersensitivity to others		−0.04	0.636	0.17	0.089	2.33	0.127
Seeking emotional distance		0.31	<0.001	0.33	<0.001	0.11	0.744
Emotional dependence on others		0.24	0.007	0.15	0.128	0.41	0.521
Reactive distancing from close friend		−0.17	0.027	−0.03	0.739	1.79	0.181
DASS	Drive for thinness	0.04	0.678	0.56	<0.001	10.12	0.001
	Body dissatisfaction	0.02	0.841	0.39	<0.001	7.65	0.006
	Bulimia	0.04	0.642	0.34	<0.001	4.11	0.043
	Perfectionism	0.26	0.003	0.36	0.002	0.42	0.518
Maintaining a clear identity	Drive for thinness	−0.37	<0.001	−0.05	0.603	7.65	0.006
	Body dissatisfaction	−0.44	<0.001	−0.27	<0.001	2.70	0.100
	Bulimia	−0.33	<0.001	−0.08	0.278	5.29	0.021
Seeking emotional distance	Drive for thinness	0.17	0.025	−0.11	0.345	4.11	0.043
	Body dissatisfaction	0.23	0.003	0.12	0.210	0.67	0.412
	Bulimia	0.08	0.403	0.21	0.029	0.88	0.347
Reactive distancing from close friend	Bulimia	0.10	0.252	0.31	<0.001	1.81	0.179
BMI	DASS	0.09	0.198	0.03	0.664	0.40	0.530
	Drive for thinness	0.38	<0.001	0.24	0.016	1.86	0.172
	Body dissatisfaction	0.34	<0.001	0.43	<0.001	0.68	0.410
	Bulimia	0.21	0.009	0.20	0.010	0.09	0.764
	Perfectionism	0.05	0.577	−0.16	0.171	2.03	0.154

The results of the model show that own emotional reactiveness and seeking emotional distance were found positively associated with DASS-21, whereas maintaining a clear identity was found negatively associated with DASS-21, with no significant differences between males and females. In addition, only among females, emotional dependence on others was found to be positively associated with DASS-21; and reactive distancing from a close friend was found to be negatively associated with DASS-21 (It should be mentioned that although these paths were significant among women only, no significant differences were found between males and females). DASS-21 was found to be positively associated with perfectionism (among both sexes) and positively associated with the drive for thinness, body dissatisfaction and bulimia among males only (with significant differences between males and females). Path analysis summary by gender is presented in Table 4.

An examination of the indirect effects (with DASS-21 as a mediator) shows that the indirect effects of own emotional reactiveness and seeking emotional distance on each of the four EDs scales was found significant among males. Among females, the indirect effects of five of the DoS scales were found significant only on perfectionism (the five scales are: own emotional reactiveness, maintaining a clear identity, seeking emotional distance, emotional dependence on others, reactive distancing from a close friend, see indirect analyses summary by sex in Table 5).

**Table 5:** Indirect analysis summary by sex.

Exogenous variables	Endogenous variables	Standardized indirect effects			
		Females		Males	
		Beta	*p*	Beta	*p*
BMI	Drive for thinness	0.00	0.480	0.02	0.663
	Body dissatisfaction	0.00	0.654	0.01	0.663
	Bulimia	0.00	0.661	0.01	0.567
	Perfectionism	0.02	0.094	0.01	0.593
Own emotional reactiveness	Drive for thinness	0.01	0.758	0.23	0.004
	Body dissatisfaction	0.01	0.890	0.16	0.005
	Bulimia	0.02	0.765	0.14	0.001
	Perfectionism	0.10	0.015	0.14	0.002
Maintaining a clear identity	Drive for thinness	−0.01	0.559	−0.08	0.081
	Body dissatisfaction	0.00	0.719	−0.05	0.095
	Bulimia	−0.01	0.643	−0.05	0.071
	Perfectionism	−0.05	0.043	−0.05	0.095
Hypersensitivity to others	Drive for thinness	0.00	0.481	0.10	0.126
	Body dissatisfaction	0.00	0.696	0.07	0.098
	Bulimia	0.00	0.671	0.06	0.058
	Perfectionism	−0.01	0.594	0.06	0.084
Seeking emotional distance	Drive for thinness	0.01	0.628	0.18	0.014
	Body dissatisfaction	0.01	0.831	0.13	0.018
	Bulimia	0.01	0.690	0.11	0.009
	Perfectionism	0.08	0.005	0.12	0.010
Emotional dependence on others	Drive for thinness	0.01	0.708	0.09	0.238
	Body dissatisfaction	0.00	0.921	0.06	0.210
	Bulimia	0.01	0.773	0.05	0.183
	Perfectionism	0.06	0.025	0.06	0.183
Reactive distancing from close friend	Drive for thinness	−0.01	0.567	−0.02	0.804
	Body dissatisfaction	0.00	0.823	−0.01	0.766
	Bulimia	−0.01	0.704	−0.01	0.775
	Perfectionism	−0.04	0.027	−0.01	0.764

**Figure 1:**
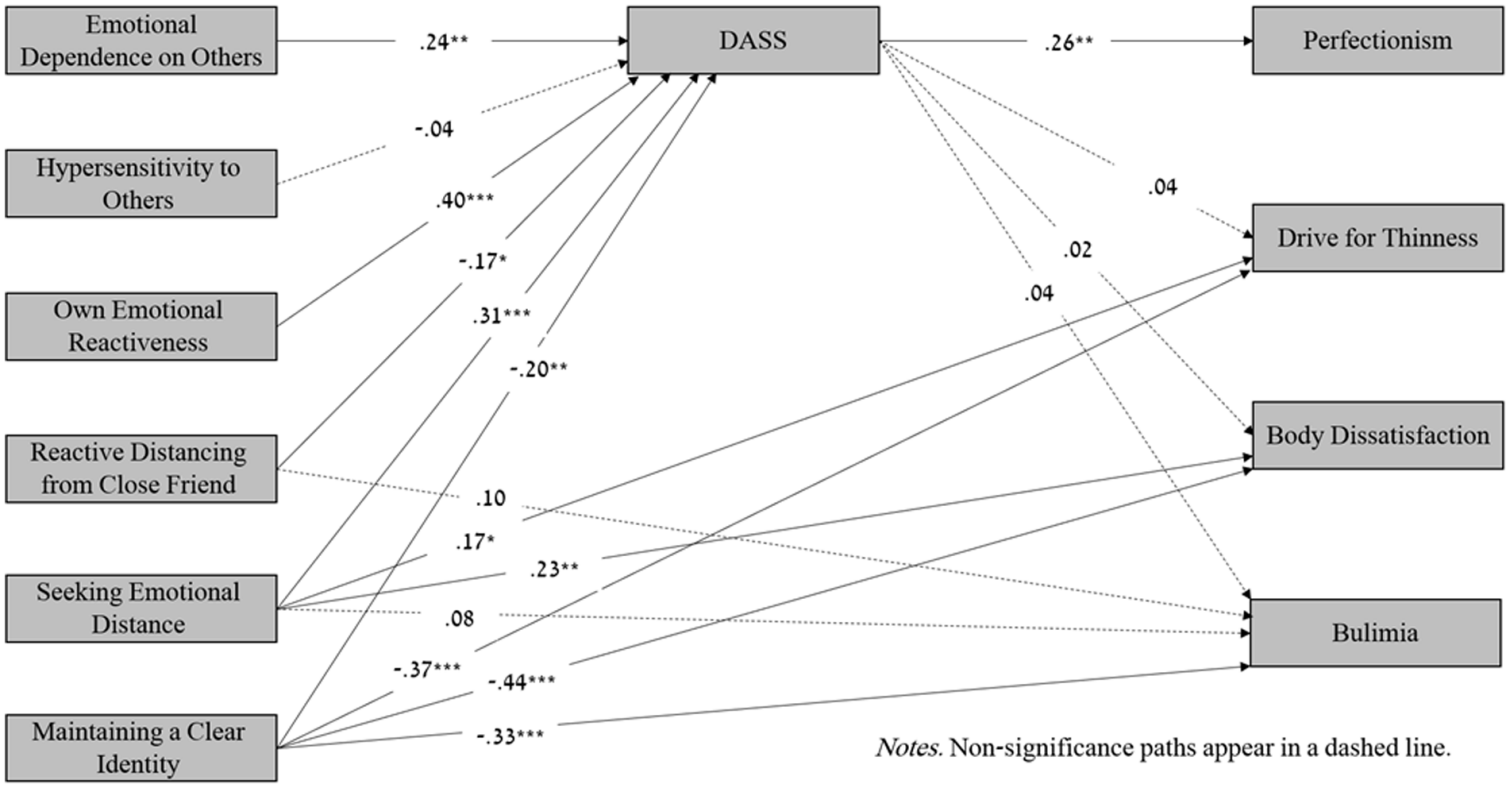
Mediation model: The associations between differentiation of self and risk of eating disorders mediated by depression, anxiety and stress (DASS) among females. Non-Significance paths appear in a dashed line.

**Figure 2:**
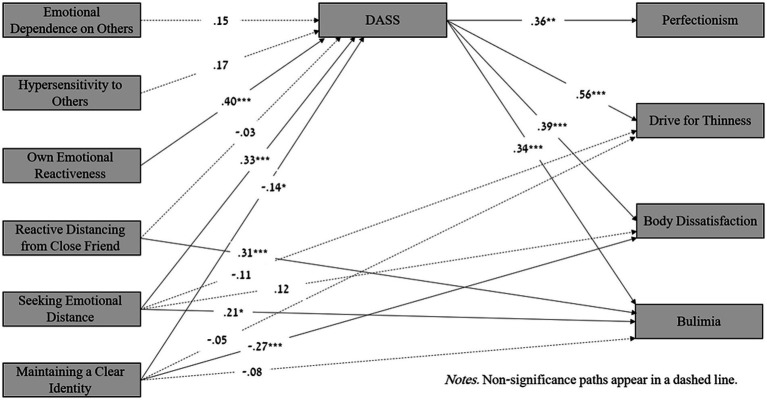
Mediation model: The associations between differentiation of self and risk of eating disorders mediated by depression, anxiety and stress (DASS) among males. Non-Significance paths appear in a dashed line.

An examination of the direct effects shows a negative direct effect of maintaining a clear identity on body dissatisfaction (for both sexes) and also on the drive for thinness and bulimia (for females only). In addition, the results show a positive direct effect of seeking emotional distance on the drive for thinness and body dissatisfaction (for females only) and on bulimia (for men only). Finally, the direct effect of reactive distancing from a close friend on bulimia was significant for males only (see Figures 1, 2).

## 5. Discussion

The present study aimed to explore the role of emotional distress as a mediator between DoS and the risk of EDs among adolescents. Due to a sex moderation effect, two mediation models were run for females and males alike. The findings indicate that emotional distress partially mediated the relationship between DoS and EDs. With regard to sex differences, female adolescents reported higher levels of emotional distress and risk of EDs than male adolescents, supporting previous studies (Doba et al., 2018). In addition, females reported higher levels of emotional dependence on others, hypersensitivity to others, and lower levels of maintaining a clear identity than males. As expected, the percentage of females at risk of EDs was higher than males.

The findings indicate that sex differences were found in the mediation model. Thus, among male adolescents, emotional distress mediated the relationships between three metrics of DoS—own emotional reactivity, seeking emotional distance and maintaining a clear identity—and all indices of the risk of EDs, while in girls, emotional distress mediated the relationships between all indices of DoS except for own hypersensitivity to others and only one measure of Eds—Perfectionism. Namely, in girls, of the four indices of EDs, only perfectionism was found to be mediated by emotional distress. A possible explanation for this result is that girls may have additional factors that increase the risk of EDs (e.g., social pressure, messages in the media regarding the ideal of beauty and thinness, etc.), whereas, for boys, it is likely that mainly emotional distress increases preoccupation with eating behaviors. The importance of this finding among adolescents emerged in light of a recent study conducted among young adults, which revealed that of the indices, perfectionism was the only one that was not found to be related to EDs or DoS (Peleg et al., 2022). Adolescents likely turn to perfectionism because they are in the process of shaping and strengthening self-identity and self-confidence. When they feel distressed or insecure, they are likely to become more perfectionists trying to improve their ego and to feel valuable. Perfectionism usually develops during childhood and adolescence, when parents play a central role (Stoeber and Childs, 2011). According to the Social Learning model (Bandura and Walters, 1977), children and adolescents develop perfectionism by observing and imitating their parents’ perfectionism. Throughout their childhood and adolescence, they are exposed to their parents’ behaviors trying to be as perfect as they are. Although perfectionism also has positive aspects in increasing motivation and responsibility (e.g., Negru-Subtirica et al., 2021), it has been reported that a high level of perfectionism may increase the risk of EDs (e.g., Peleg et al., 2016; Johnston et al., 2018). This topic is of great importance and merits further investigation.

In addition to the relationships mediated by emotional distress, direct relationships have also been found between DoS and the risk of EDs. Thus, in girls, seeking emotional distance and maintaining a clear identity significantly affected the metrics of risk of EDs (except for perfectionism). Similar results were found among male adolescents: seeking emotional distance, maintaining a clear identity, and reactive distancing from a close friend directly affected all metrics of risk of EDs (apart from perfectionism). The current results support recent studies indicating poor DoS in adolescents with a high risk of EDs (e.g., 10) and adverse relationships between DoS and emotional distress. In addition, the results strengthen the Family Systems Theory (Kerr and Bowen, 1988), which suggests that DoS is rooted in early family experiences and affects the individual’s ability to deal with stressful situations. Moreover, this is one of the few studies examining DoS in adolescents. Since DoS can still change at this point, understanding its impact on mental and physical health is critical and allows for preventing the deterioration of emotional difficulties, including the risk of EDs.

To sum up, the present findings partly support the hypothesis that emotional distress mediates the relationship between DoS and EDs. The results suggest that individuals with a high risk of EDs have difficulty dealing with stressful situations and social pressures due to low DoS. It can be speculated that adolescents who find it challenging to maintain their self, identity, and beliefs tend to feel upset and distressed because the responses of significant others may threaten them, and as a result of the heightened emotional distress, they increase perfectionistic behaviors and are at a higher risk of EDs.

## 6. Limitations

The results should be treated with caution due to several limitations. First, given the correlational nature of the study design, it is not possible to assume causality. Indeed, the option that EDs may lead to low DoS is less likely. Nonetheless, longitudinal research is needed to offer more comprehensive information on the suggested relationships between DoS, emotional distress, and EDs. Second, the current study did not examine other aspects of EDs (e.g., social pressure, body image, self-confidence, social network use, etc.). Third, the sample in the current study is not representative. We therefore suggest that further investigations of this issue use a larger representative sample of teenagers from different regions and ethnic groups and include additional variables (e.g., social media use, body image).

## 7. Conclusion

Notwithstanding the limitations, the present study deepens the understanding of risk factors of EDs and the possibility that family and emotional factors are involved in the etiology of EDs among adolescents. In addition, while the relationship between emotional distress and the risk of EDs is well documented, the present study is novel. It indicates that high DoS may reduce emotional distress, which in turn may reduce the risk of EDs. Another strength of the present study lies in the investigation of two separate models for males and females alike, which enables an in-depth understanding of the risk factors that characterize males and females.

## 8. Clinical implications

The findings may have significant applied contributions. It is reasonable to assume that promoting the healthy development of children and adolescents will enable adaptive individuation (Gilmore and Meersand, 2013) and thus decrease emotional distress. For this purpose, we suggest planning workshops for teenagers, tailored to each sex separately, which deal with emotional distress by improving DoS and direct communication and thus helping to reduce the risk of EDs. For example, boys could be helped to improve their emotion regulation and to share their emotions instead of repressing them, while girls could be helped to work on their tendency toward perfectionism. These workshops should be able to prevent escalation and deterioration during adolescence, which is a critical age for the outbreak of EDs. We also recommend holding workshops for parents to provide them with coping skills aimed at improving DoS and relationships with their children.

## Data availability statement

The raw data supporting the conclusions of this article will be made available by the authors, without undue reservation.

## Ethics statement

The studies involving human participants were reviewed and approved by The College Institutional Review Board approved the complete study protocol on 9.12.2019 (EMEK YVC 2020–13). Written informed consent to participate in this study was provided by the participants’ legal guardian/next of kin.

## Author contributions

OP constructed the research plan, chose the research tools, and was responsible for data collection, analysis, and interpretation of the data, and was a significant contributor to writing the manuscript. MB-N was a significant contributor in interpreting the data and writing the manuscript. OT helped in interpreting the data. All authors contributed to the article and approved the submitted version.

## Funding

This work was supported by Max Stern Yezreel Valley College’s research grant.

## Conflict of interest

The authors declare that the research was conducted in the absence of any commercial or financial relationships that could be construed as a potential conflict of interest.

## Publisher’s note

All claims expressed in this article are solely those of the authors and do not necessarily represent those of their affiliated organizations, or those of the publisher, the editors and the reviewers. Any product that may be evaluated in this article, or claim that may be made by its manufacturer, is not guaranteed or endorsed by the publisher.
